# Design and Near-Infrared Actuation of a Gold Nanorod–Polymer Microelectromechanical Device for On-Demand Drug Delivery

**DOI:** 10.3390/mi9010028

**Published:** 2018-01-13

**Authors:** John Jackson, Aurora Chen, Hongbin Zhang, Helen Burt, Mu Chiao

**Affiliations:** 1 Faculty of Pharmaceutical Sciences, University of British Columbia, 2045 Wesbrook Mall, Vancouver, BC V6T 1Z3, Canada; auroraonmusic@gmail.com (A.C.); helen.burt@ubc.ca (H.B.); 2 Department of Mechanical Engineering, University of British Columbia, 6250 Applied Science, Vancouver, BC V6T 1Z4, Canada; izhanghongbin@gmail.com (H.Z.); muchiao@gmail.com (M.C.)

**Keywords:** microelectromechanical systems (MEMS), near infrared (NIR) laser actuation, drug-release device, docetaxel, nanorod

## Abstract

Polymeric drug delivery systems usually deliver drugs by diffusion with an initial burst of release followed by a slower prolonged release phase. An optimal system would release exact doses of drugs using an on-demand external actuation system. The purpose of this study was to design and characterize a novel drug-delivery device that utilizes near infrared (NIR 800 nm) laser-actuated drug release. The device was constructed from biocompatible polymers comprising a reservoir of drug covered by an elastic perforated diaphragm composed of a bilayer of two polymers with different thermal expansion coefficients (ethylenevinylacetate (EVA) and polydimethylsiloxane (PDMS) containing gold nanoparticles). Upon illumination with a NIR laser, the gold nanoparticles rapidly heated the bilayer resulting in bending and a drug-pumping action through the perforated bilayer, following sequential laser-actuation cycles. Devices filled with the anti-proliferative drug docetaxel were seen to release only small amounts of drug by diffusion but to release large and reproducible amounts of drug over 20 s laser-actuation periods. Because NIR 800 nm is tissue-penetrating without heating tissue, suitable geometry drug-delivery devices might be implanted in the body to be actuated by an externally applied NIR laser to allow for on-demand exact drug dosing in vivo.

## 1. Introduction

Implantable drug-delivery systems may be used to deliver drugs either systemically or locally. Systemic delivery systems might be implanted intramuscularly (e.g., Nexplanon™ to deliver the hormone contraceptive progesterone) whereas local systems are implanted next to disease sites (e.g., Gliadel™ wafers to deliver carmustine to treat brain tumors). Implantable systemic systems offer the advantage of controlled release from a single administration, whereas local delivery has advantages of controlled release and high local concentrations with low systemic toxicity [1,2,3,4]. 

Over the last 30 years, numerous polymer-based drug delivery systems such as injectable pastes, films, or microspheres have been developed to facilitate the controlled release of drugs [5,6,7,8]. However, these systems rely on diffusion-based release, which is nearly always characterized by an initial burst phase of release followed by a slower sustained phase that reduces with time. Such release profiles may be acceptable for treating cancerous tumors [6] but not for treating delicate tissues such as the retina (e.g., in diabetic retinopathy) where high local drug concentrations might permanently damage both normal cells and pathogenic cells. Clearly, in an ideal world, an implantable drug delivery device might deliver an exact dose of drug either at predetermined intervals or by external actuation on demand by a clinician. 

Recently, a number of implantable systems have been described that provide such on-demand performance. These stimulated groups of delivery systems include controlled-release devices that involve materials, mechanical design and fabrication techniques, such as devices based on microelectromechanical systems (MEMS) and nanoelectromechanical systems (NEMS) [9,10,11,12]. The devices consist of different functional components such as micropumps, microvalves, reservoirs, channels, etc. forming an integrated system for achieving more sophisticated and precise drug delivery [10,13]. Release mechanisms may use mechanical (e.g., squeezing) electrical, electrothermal or magnetic methods. 

In this lab, magnetic methods have been pursued because they involve the simple application of an external magnet to actuate the device [14,15,16,17,18]. These devices comprise a poly dimethyl siloxane (PDMS) MEMS drug storage reservoir manufactured in various shapes using a pattern-transfer approach called soft lithography, which refers to a group of techniques using elastomeric stamps and molds [19]. The drug is then pumped out of the reservoir by stimulation and deflection of an elastic magnetic diaphragm or sponge [16,17] next to the drug reservoir. One limitation of magnetic systems is that the degree of deflection of the diaphragm or sponge (and hence the dose of drug released) may be dependent on the strength, proximity and angle of the actuating external magnet. 

A different approach to achieving diaphragm deflection in a MEMS drug-delivery system, similar to those magnetic systems [14,15,16,17,18], might be to replace the magnetic diaphragm with an elastic membrane that might deflect under near infrared (NIR) illumination. Such a system is highly novel because this is the first description to our knowledge of the assembly and operation of a small device containing a drug reservoir, a flexing bilayer diaphragm membrane, incorporation of gold nanorods, and actuation by an external laser. Lasers that operate in the NIR region are known to penetrate tissues well and the NIR wavelength range (700–900 nm) lies above the absorption wavelength for biological molecules and below that of water, allowing little laser attenuation or tissue damage [20]. This system sidesteps the need for any onboard power or electronics and is less limited by distance-related actuation attenuation seen in magnetic systems. 

The core technology in this report describes the assembly and functioning of a bilayer polymeric membrane composed of polymers with different expansion coefficients and one membrane containing dispersed gold nanoparticles. The mechanism of actuation was proposed to be via illumination of the membrane with NIR laser light so that Plasmon resonance and instant heating of the gold nanoparticles [20,21] might allow for heating of the bilayer and strong bending based on different degrees of expansion of the two polymers (like a metal bilayer thermocouple). The manufacture, assembly and effective functioning of a drug-loaded device is also described. 

## 2. Material and Methods

### 2.1. Design

**Figure 1:**
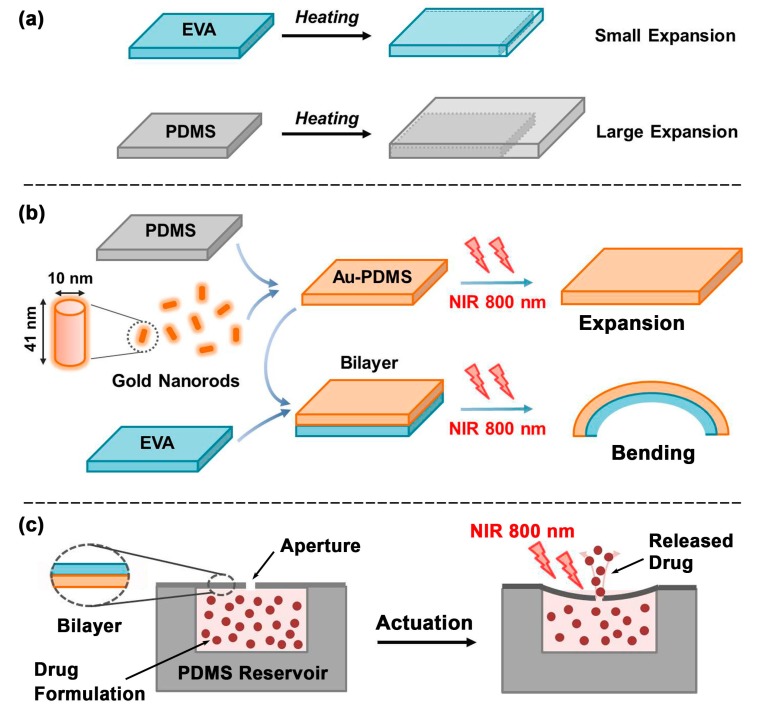
Schematic of device design and operation, (a. principle of expansion, b. principle of bending, c. principle of drug release).

The design of this device features a simple reservoir-pump system with a thermally sensitive bilayer membrane. The device consists of a reservoir space for drug storage sealed by a thin bilayer membrane that acts as a pump when actuated. The thin bilayer membrane contains a small aperture in the center. Upon actuation, the thin membrane warms up and deflects, resulting in the drug being forced out of the reservoir. When the thin membrane cools down, the membrane returns to its original state and generates negative pressure within the reservoir, which then refills from the surrounding drug-poor liquid. The schematic in Figure 1 illustrates the design and operation of the device. 

In order to generate the pump motion, the thermally sensitive bilayer membrane is made of two polymers with different thermal expansion coefficients (TECs) that deflects when heated up. Timoshenko’s model [22] was used to estimate the deflection of the bilayer membrane composed of polymers with mismatched thermal expansion coefficients. The temperature elevation in the thin bilayer membrane generates in-plane strain that forces the membrane to bend, and as a result pumps drug out of the reservoir. As shown in equation below, the curvature *κ* is expressed as a function of differences in the thermal expansion coefficient, temperature change, Young’s modulus and thickness of each material.

(1) $$\kappa =\frac{6\left(h_{a}+h_{b}\right)\Delta \alpha T}{nmh_{a}{}^{2}+4h_{a}{}^{2}+4h_{b}{}^{2}+6h_{a}h_{b}+\;\frac{1}{m}\frac{1}{n}h_{b}{}^{2}}$$

where: *κ* is the curvature; *T* is the temperature; $h_{a}$ is the thickness of the top layer; $h_{b}$ is the thickness of the bottom layer; Δα is the difference in thermal expansion coefficient of top and bottom; m is $h_{a}$/$h_{b}$; n is $E_{a}$/$E_{b}$; $E_{a}$ is the Young’s modulus of the top layer; and $E_{b}$ is the Young’s modulus of the bottom layer.

This equation suggests that with selected material and device geometry, the larger the difference in the TEC of the polymers and the bigger the temperature change, the larger the degree of deflection. 

The two polymers used were poly dimethyl siloxane (PDMS) and ethylene vinyl acetate (EVA), both of which are elastic and have quite different expansion coefficients. The TEC of PDMS is 3.0 × 10^−4^ °C^−1^ and the TEC of PEVA is 1.6 × 10^−4^ –2.5 × 10^−4^ °C^−1^.

### 2.2. Fabrication

#### 2.2.1. Poly Dimethyl Siloxane (PDMS) Reservoir

The fabrication of the PDMS reservoir was carried out as follows: the PDMS reservoir body and base were fabricated separately and sealed together through plasma bonding. PDMS (Sylgard 184 Silicone Elastomer, Dow Corning Corporation, Midland, MI, USA) was mixed together at 1 part crosslinking agent to 5 parts PDMS monomers for 3 min before degassing for 2 min. The mixture was poured into two petri dishes (reservoir body and base respectively), and placed in an oven at 70 °C for 2 h to cure. A square-shaped reservoir body with a hollow center was carefully carved out using a surgical knife and an 8 mm-diameter puncher. Another matching square was carved out of thick material using a surgical knife to comprise the base of the reservoir. Both reservoir body and base were treated with air-plasma for 1 min and 20 s at ∼700 m Torr chamber pressure. The two treated surfaces were firmly pressed together to form a permanent air-tight bond between the reservoir body and bottom. The reservoir was then placed in a 70 °C oven for 20 min to further enhance the bonding.

#### 2.2.2. Au-PDMS Membrane

A PDMS solution (1:5 ratio) was prepared. A gold nanorod solution was prepared by mixing 3 wt % gold nanorods (NanoPartz Inc., Loveland, CO, USA, 10 nm diameter, 41 nm length, 808 nm excitation wavelength) with ethanol as solvent. The Au-ethanol solution was mixed with 1:10 PDMS for 10 min followed by 3 min degassing to ensure proper mixing. The mixture was then placed in an oven at 60 °C for 4 min to allow any residual ethanol to evaporate completely*.* This method gives a 0.3% w/w gold in PDMS membrane. 

The resulting Au-PDMS viscous liquid was degassed again for 1 min before it was poured on to a glass slide that was pre-coated with 20% polyacrylamide (PAA) used as a sacrificial layer. This liquid was then spin-coated on to PAA sacrificial layer-coated glass slides using three steps: 10 s spinning at 500 rpm, followed by 30 s spinning at 1200 rpm, then 30 s spinning at 1700 rpm. The gradual spinning approach ensures uniform coating of the glass slide using viscous Au-PDMS pre-cured liquid. The Au-PDMS coated glass slide was cured on a hot plate at 150 °C for 15 min.

#### 2.2.3. Thin Bilayer Membrane

Both the ethylenevinylacetate (EVA) and Au-PDMS layers must be pre-prepared separately before joining into the thin bilayer membrane. The EVA layer was made by first dissolving 124 mg of solid EVA beads (Polysciences, Warrington, PA, USA) in 20 mL dicholoromethane (DCM) overnight. Because of the high volatility of DCM, the solution was chilled in a refrigerator before handling. 8 mL of the EVA solution was poured into a Teflon-coated petri dish (Welch Fluorocarbon Inc., Dover, NH, USA) and left to evaporate in the refrigerator overnight to form 25 µm thickness EVA membranes.

Once the EVA layer was ready and with the previously prepared Au-PDMS membrane on the glass slide, a thin bilayer membrane was made by bonding them together through surface treatment with trifunctional saline 3-aminopropyltriethoxysilane (APTES, Sigma-Aldrich, Oakville, ON, Canada). The EVA layer was soaked in 1:1:2 volume ratios of H_2_O_2_, HCl, and H_2_O, respectively, for 10 min. The solution was removed, and the EVA layer was washed gently with a small amount of deionized water. Meanwhile, a 2% APTES solution was prepared by mixing 0.2 mL APTES with 10 mL of 35% ethanol. The EVA layer was soaked in the 2% APTES solution for 40–60 min, rinsed with a small amount of deionized water, and gently air-dried. Both the surface-modified EVA layer and the Au-PDMS from the previous step were air-plasma treated for 80 s to bond them together permanently. 

#### 2.2.4. Reservoir Loading with Drug

A solution of docetaxel (DTX, Polymed T, Houston, TX, USA) (40 mg/mL) was prepared by dissolving DTX in a 50/50 mix of ethanol and DCM solvent. This solution was doped with 10 μL of 3H tritium labeled docetaxel (1 uCi/μL) (Moravek, Brea, CA, USA). Both the PDMS reservoir and bilayer thin membrane were air-plasma treated before radio-labeled DTX solution was gently pipetted (5 μL) into the reservoir space and solvent evaporated by placing the loaded device in a 60 °C oven for 2 min to evaporate. This filling was repeated 2 times to provide 0.4 mg of drug in the reservoir. Deposition of drugs and evaporation of excess solvent should take no longer than 10 min to ensure successful surface bonding. The bilayer was then bonded to the reservoir. Theoretically a reservoir of this size might hold approximately 10 mg of drug but diaphragm function might be compromised with such payloads.

#### 2.2.5. Laser Drilling

**Figure 2:**
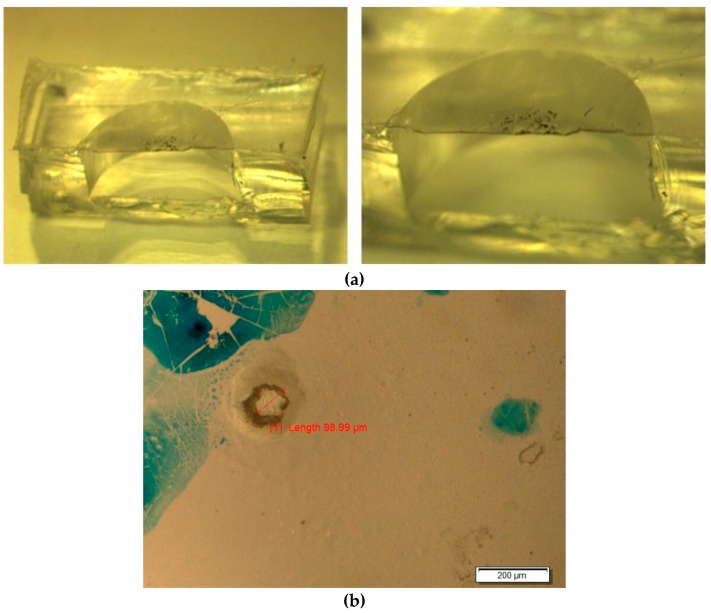
(**a**) Cross-sectional view of a bisected device. (**b**) Microscopic view of aperture on bi-layer membrane.

A Nd:YAG laser (Quicklaze, New Wave Research, Sunnyvale, CA, USA) was used to ablate the aperture in the bilayer thin film. The parameters used were as follows: Green laser (532 nm wavelength), 100% power, laser pulses at 40 Hz. Care was taken to ensure the laser beam did not reach the bottom of the reservoir where the radioactive DTX was deposited. The aperture was examined under a microscope before proceeding to the next step. A cross-sectional view of a scalpel-bisected device is shown in Figure 2. A ~100 um aperture may be seen in the overhead microscopic view in Figure 2b (the blue marks show laser alignment markings).

#### 2.2.6. Reservoir Filling for Drug-Release Measurements

The assembled device was transferred to a glass vial filled with 10 mL of 0.2 μm filter-sterilized Phosphate-buffered saline (10 mM pH 7.4 PBS) containing 4% bovine serum albumin (BSA) (Sigma-Aldrich). Due to the hydrophobic nature of PDMS and considering how small the laser-drilled aperture was, in order to replace the air inside of the sealed reservoir space the device was placed in vacuum while being held under water. Care was taken to monitor the intactness of the membrane as well as the remaining air space in the reservoir. Once most of the air was gone, the vacuum chamber was gently depressurized to allow the PBS-4% BSA solution to refill the reservoir space. The device was left immersed in the same solution for 2 days to allow the remaining air space to be completely filled.

#### 2.2.7. In Vitro Drug-Release Studies

Once the reservoir space was filled with PBS-4% BSA solution the device was considered ready for the drug-release experiment. The setup included an optical bench consisting of a 1.5 W 800 nm laser, 10 mL in PBS-1% BSA solution, a 1 cm-thick ring base, and a small petri dish to hold the sample device. The purpose of the study was to measure the amount of drug released from the device after being actuated by a 1.5 W 800 nm laser for a certain amount of time.

A ring base was placed underneath the 1.5 W 800 nm laser beam so the petri dish and the sample could be placed above it in the hollow area to avoid heat transfer from the substrate. The device was primed by placing it under the laser beam for 20 s followed by a 40 s cooling period and repeating this 25 times to allow the drug release to be stabilized. Once priming was completed, a droplet of 30 µL 1% BSA in PBS solution was deposited on the surface of the membrane directly on top of the reservoir space and covering the aperture. The device was then placed under the laser beam with the laser beam overlapping the membrane on top of the reservoir space. The device was actuated for 20 s before another droplet of 30 µL of PBS-1% BSA solution was gently added to the top of the membrane. The device was then removed from the laser beam to cool down for 40 s. During this 40 s cooling the reservoir will take up PBS-1% BSA solution as the diaphragm returns to its original position under cooling so that no diffusion will occur and this period was not considered part of the release or diffusion cycle. Adding the second droplet of PBS solution mechanically stirs the original droplet and allows the drug released to be mixed properly in a short period of time so that when the membrane restores and draws liquid back into the reservoir less drug will be returning to the device, which mimics the biological environment in the human body. After cooling for 40 s, ~60 µL of liquid was carefully collected from the surface of the membrane and transferred into a scintillation vial for drug-release quantitation. The device was then left for 4 min. Background diffusion was measured after one drug-release count was collected. The membrane was rinsed with PBS solution three times to remove any residual drug. A droplet of 30 µL of PBS-1% BSA solution was deposited and left on the membrane for 4 min without actuation. After 4 min, the droplet was collected in a scintillation vial for the background diffusion count.

#### 2.2.8. Thermal Data Collection

A thermaCAM E320 FLIR thermal imaging camera (FLIR Systems Wilsonville, OR, USA) with a resolution of 320 × 240 was used to record the temperature of the sample membrane when it was exposed to the laser beam at fixed power: 1.5 W 800 nm. PDMS membranes (containing gold nanoparticles) attached to a glass slide were used for these studies. The laser beam was focused to 6 mm diameter so it aligned perfectly with the sample membrane. When underwater thermal data was collected, the laser beam, sample and thermal camera were pre-aligned before the sample was immersed completely under a small amount of water in order to achieve the same experimental conditions. The membrane was exposed to NIR for increasing times and the temperature of the membrane recorded. 

#### 2.2.9. Deflection Measurements

Since the membrane deflects inwards, to visualize deformation of the membrane an empty device (unloaded) was cut using a scalpel in half on the *X*–*Z* plane or the *Y*–*Z* plane to expose half of the reservoir and membrane. The half device was carefully matched for half of the laser beam during actuation. Membrane deformation was observed and recorded using a microscope with micrometer, which was carefully adjusted to the horizontal view of the device. Meanwhile, temperature was recorded using the FLIR thermal imaging camera.

Since the TEC of PDMS is larger than the TEC of EVA, one might anticipate that when these two membranes are bonded together, elevation of temperature will result in the membrane deflecting in such a way that the PDMS layer forms the outer part circle and the EVA layer forms the inner part circle. The fabrication design includes interfaces of EVA (top layer membrane)–PDMS (bottom layer membrane) and PDMS (bottom layer membrane)–PDMS (reservoir) so that, on heating, the membrane deflects downwards. 

It was noted that when the membrane was cut in half, its boundary condition and geometry changed because not all sides of the membrane are constrained by the reservoir edges after cutting. Therefore, direct measurement of deflection might differ from the actual deflection in an intact device. To solve this problem, a new batch of devices was fabricated with the composition of the thermal-responsive membrane reversed so that the interfaces from top to bottom are PDMS–EVA and EVA–PDMS. This way, when the membrane is heated, it undergoes a convex deformation instead of a concave one, so the deformation could be directly measured through a microscope.

## 3. Results

### 3.1. Characterization

#### 3.1.1. Overall Device Characteristics

The manufacturing methods resulted in the production of a robust device where the bilayer was free of imperfections and the bond between the layers was strong, as witnessed by no separation under heavy manipulation. 

**Figure 3:**
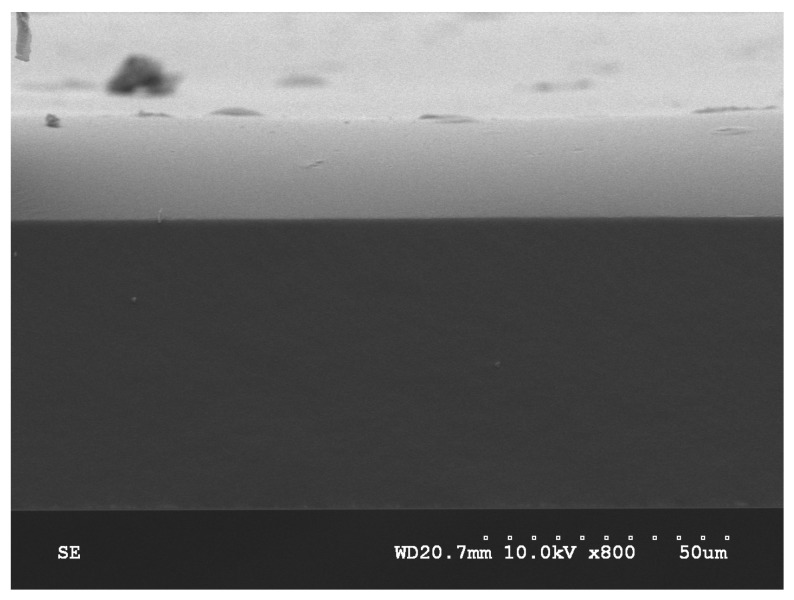
Scanning electron microscope (SEM) image of the bilayer showing the thinner lighter-colored ethylene vinyl acetate (EVA) membrane on top and the darker polydimethylsiloxane (PDMS)-gold nanoparticle membrane with an approximate thickness ratio of 1:3 (EVA:PDMS).

A bisected device is shown in the optical image in Figure 2. The aperture may be seen in the overhead optical microscope photograph Figure 2b. The aperture’s size is approximately 100 μm and the blue markings are residual laser-targeting reference points. The hollow reservoir covered by a thin membrane can be clearly seen. The scanning electron microscope (SEM, Hitachi S4700, Schaumburg, IL, USA) image in Figure 3 shows the thinner light-colored EVA membrane with the darker thicker bottom layer being PDMS containing gold nanorods (Au-PDMS). The membrane thickness was measured at 81 μm. 

Timoshenko’s model [22] was used to estimate the deflection of the bilayer membrane composed of polymers with mismatched thermal expansion coefficients. This model predicts the optimal thickness ratio between EVA and Au-PDMS to achieve maximum deflection in a free bounding condition to be between 1:3 and 1:5. This SEM image indicates the sample yielded an approximate 1:3 thickness ratio between EVA and Au-PDMS for optimal operation as a diaphragm pump membrane. 

#### 3.1.2. Thermal-Heating Measurements

**Figure 4:**
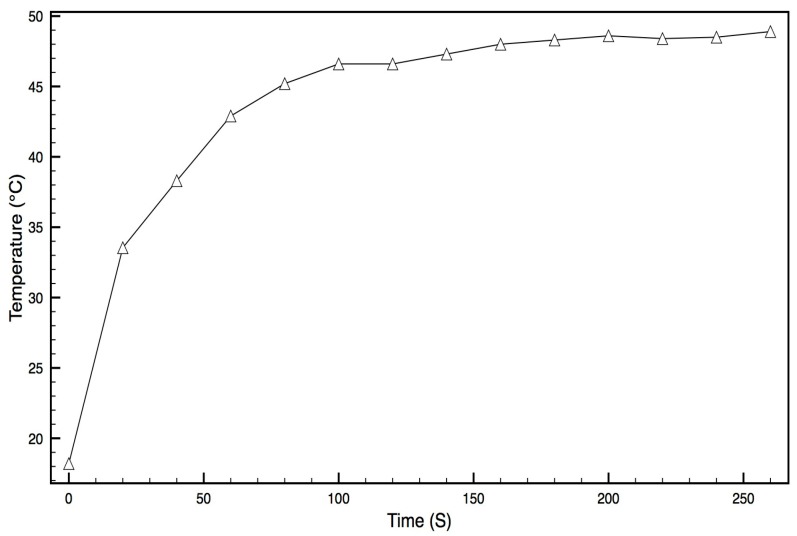
Temperature increase in a PDMS–gold nanoparticle membrane held under water with continuous illumination by the near infrared (NIR) laser.

The Au-PDMS membrane on a glass slide was placed under water and illuminated with NIR laser light. At defined times, the temperature was recorded under this illumination. The temperature of the membrane rose quickly in the first 25 s and then slowed down before reaching a steady state of 50 °C by 2 min. At this time it is likely that any further temperature increase was offset by cooling losses to the water. These data clearly demonstrate effective heating of the Au-PDMS membrane under NIR laser light, as shown in Figure 4. 

**Figure 5:**
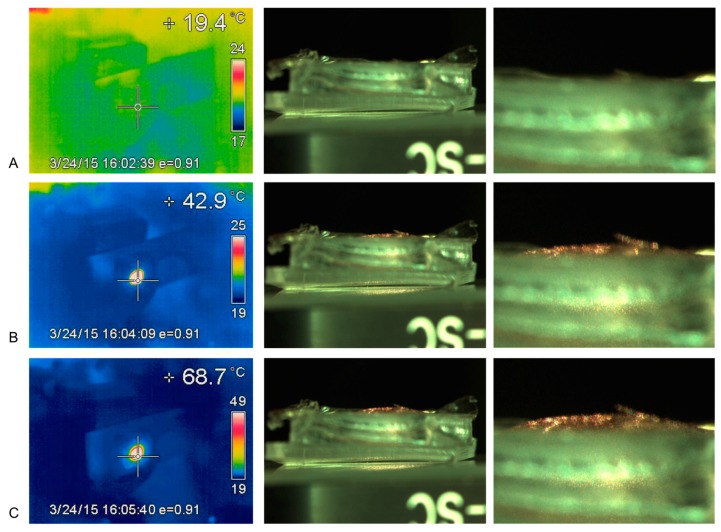
Thermal heating and convex (upwards) bending of a reversed bilayer (PDMS uppermost and EVA lower) Reversal was used to allow quantitation of deflection. Left hand panels show temperature in centre of bilayer, Middle panels show upwards bending and right panels show zoomed image of the top of the bilayer membrane, ((**A**) 19 °C, (**B**) 43 °C and (**C**) 68 °C).

**Figure 6:**
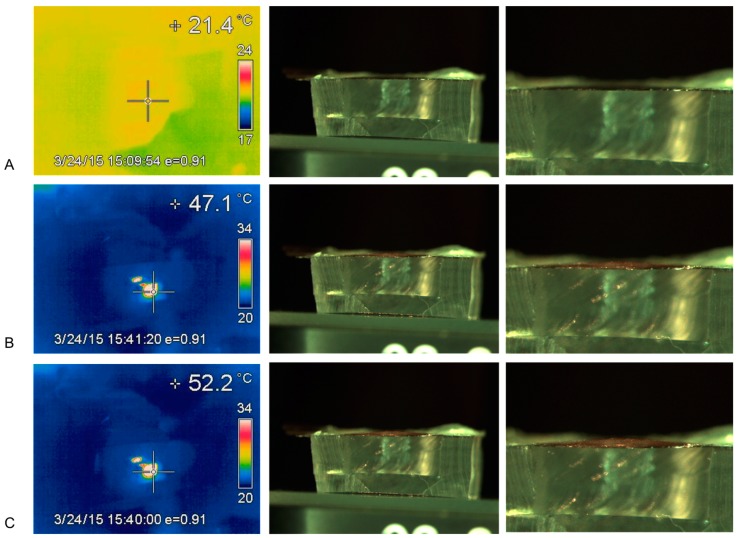
Thermal heating and concave (downwards) bending of a correctly oriented bilayer (PDMS lower and EVA upper) Left hand panels show temperature in centre of bilayer, Middle panels show bending and Right panels show zoomed image of the top of the bilayer membrane, ((**A**) 21 °C, (**B**) 47 °C and (**C**) 52 °C).

When assembled devices containing bilayer membranes were exposed to NIR laser light, the membranes quickly rose to high temperatures in the absence of water. This was observed in experiments measuring membrane deflection under NIR laser light (Figure 5 and Figure 6) where temperatures as high as 69 °C were observed. These data further support the functional heating of the diaphragm part of a device under NIR laser light.

#### 3.1.3. Temperature vs. Membrane Deflection

When exposed to NIR laser light the membrane on the device quickly heated up and also deflected, as seen in Figure 5 and Figure 6. In this experiment the bilayer was assembled in the reverse orientation so that the membrane deflected upwards (convex) and could be measured using the microscope. The upwards movement of the membrane as a function of temperature can be observed in Figure 5 so that at 68.7 °C (right-hand caption) a convex dome can be seen above the device. A normally assembled device (EVA membrane on top and Au-PDMS membrane fixed to reservoir) was also observed under NIR laser exposure. The membrane deflected downwards (concave manner) but the deflection could not be quantitated because the microscope was unable to focus on the membrane through the reservoir body.

The bilayer heating and downward deflection of a normal-oriented bilayer can be visualized in Figure 6. 

**Figure 7:**
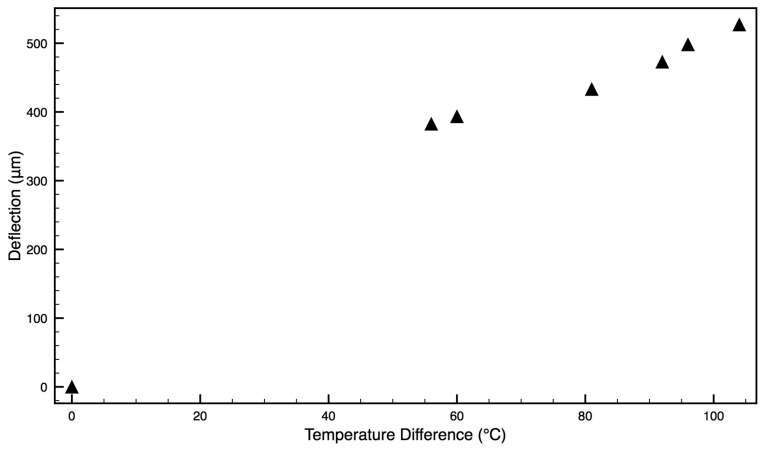
The deflection of a diaphragm membrane on an assembled device oriented in the reverse direction (PDMS-gold upper) as a function of bilayer temperature.

Because the membrane heats rapidly under dry conditions, with no heat losses to water, it was impossible to measure deflections at lower temperatures. At higher temperatures the degree of membrane deflection was proportional to the heating temperature, as seen in Figure 7. 

These observations confirm the basic working principles of the device, namely that the NIR laser causes a rapid heating of the diaphragm and this heating translates to a membrane deflection above a device that might be used as a pumping action. 

### 3.2. Docetaxel (DTX) Drug Release

#### In Vitro Drug-Release Study

Laser-actuated DTX release and background diffusion from three devices (A, B and C) was studied over a period of 3 days. In these drug-release experiments, the membrane temperature rose from 20 °C (room temperature) to 40 °C after 20 s actuation using a 1.5 W 800 nm laser. 

**Figure 8:**
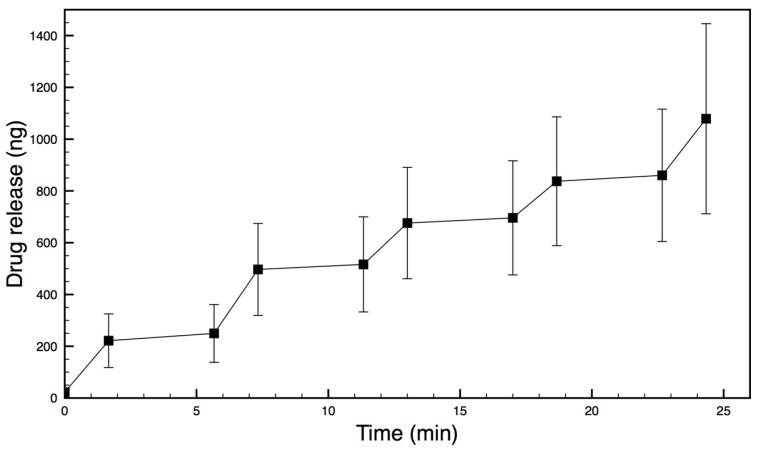
Release of docetaxel from Device A following 5 actuation cycles starting at *t* = 0 min. The step phase of release happens during laser actuation and is followed by 4 min (background diffusion) with the laser turned off. The experiment was repeated on three separate days, and each point is the mean of those points.

The drug-release data from each daily experiment for each device was then pooled. Results were averaged at matching activities (first actuation to first actuation, first diffusion to first diffusion etc.) and shown in Figure 8 for Device A, including errors. 

**Figure 9:**
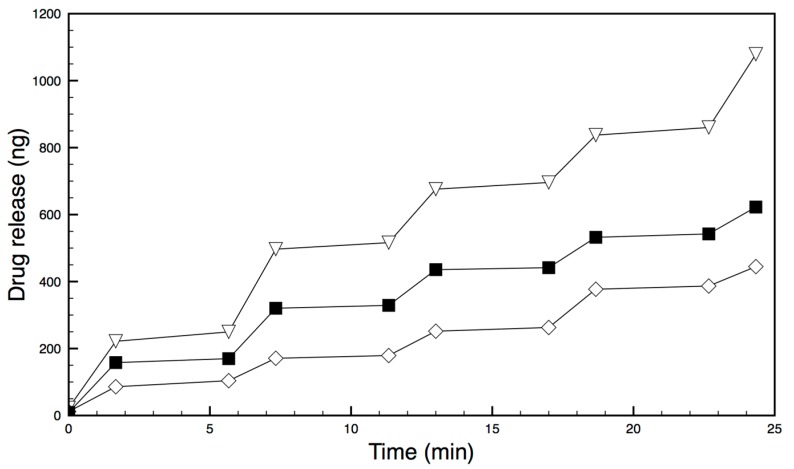
Release profiles for three different devices over a 21 min period. The step phase of release happens when the laser is actuated followed by 4 min of diffusion.

Each of the three devices (A, B and C) successfully released the drug on demand when illuminated with laser light, as seen in Figure 9 (errors not shown for clarity). The amount of drug released at each actuation was very reproducible and shown in Table 1 as the rate of drug release per minute. Each device released between approximately 50 ng and 120 ng of drug/min per actuation cycle, with between only 2–6 ng being released during diffusion (non-laser actuated) parts of the experiment. All three samples exhibited similar release profiles. Each actuation cycle releases approximately 20 times more drug/min than occurs during the diffusion cycle. These amounts represent less than 0.025% of the reservoir drug payload. Drug-loaded devices were also manufactured using membranes composed of a bilayer that was gold-free as well as devices containing only a gold-loaded PDMS monolayer (no bilayer). These devices were actuated many times with the laser, or left (diffusion leakage), and drug release was measured. Under laser illumination these devices did not release any drug above background diffusion levels. 

## 4. Discussion

The design and manufacture of externally actuated MEMS drug-delivery devices has been previously described by many groups [14]. In particular, magnetically actuated devices made from PDMS have been the focus of work described by Chiao’s group [14,15,16,17,18]. These devices include basic pilot device manufacture to working devices ready for implantation into target tissues. These working devices include ocular systems designed for attachment to the back of the eye and micro-cylindrical devices for needle insertion into the prostate [17,18]. Both these working devices used the drug docetaxel, which is a potent anti-proliferative drug used to treat ocular and prostatic-type proliferative diseases. This drug is particularly suited to use in these devices because the solubility of the drug is low (less than 100 μg/mL in aqueous media) so only a small amount of drug in the reservoir is used up in each actuation delivery cycle, ensuring a durable drug supply. 

In this study, the effective manufacture of a similar PDMS reservoir-diaphragm pump has been described. However, although the reservoir geometry, manufacture and drug type are similar to previous devices, the basic actuation mechanism is entirely different. Other workers have described bilayer bending as a possible actuation method [22] and the incorporation of gold nanoparticles in polymer membranes [23]. However, the polymeric bilayer-bending mechanism and the thermal actuation of onboard gold nanoparticles by a NIR laser is entirely novel and represents a uniquely different approach to pump actuation from magnetic systems. There were numerous technically challenging aspects to the assembly of these working devices. These include the homogenous distribution of gold nanoparticles (nanorods) in the thin PDMS membrane, the successful bonding of a second thinner EVA membrane to the PDMS membrane, and the bonding of the bilayer membrane to the reservoir body. These technical difficulties were overcome as observed in Figure 2 and Figure 3, and the successful operation (heating, flexing, drug release) illustrated in Figure 4, Figure 5, Figure 6, Figure 7, Figure 8 and Figure 9. Berry KR et al. [23] described the dispersion of gold nanoparticles in PDMS membranes and showed a concentration-dependent uniform pink coloration of the membrane. We used the same dispersion method and observed the same increasing uniform coloration as we increased the concentration of nanorods, demonstrating the uniform dispersion of gold nanorods in our membranes. 

A number of drug-loaded devices were successfully manufactured using these methods, producing robust systems that could be hand manipulated without disassembly or failure. 

Using magnetic actuations with similar PDMS-based reservoir devices containing 100 μm apertures, we have previously shown rapid and reproducible drug release using short actuation times [15,16,17]. The main reason for not increasing the aperture size in this study was to ensure low levels of background diffusion. Using 100 μm apertures, we have found very low levels of drug diffusion occur in the non-actuation phase [15,16,17] which is a critical aspect of an on-demand drug-release system. Furthermore, if the aperture size were reduced the device might require unwanted longer laser exposure and relaxation times with little pay off in terms of reduced background diffusion. 

There are previous reports of polymer-coated gold nanoparticles being used for medical and pharmaceutical purposes [24,25,26,27,28]. Such methods include systemic delivery for targeting or local injection and NIR laser actuation for the thermal ablation of cancer tissue. Interestingly, Croissant J [27] and Li H [28] used lasers to heat gold nanoparticles to activate the release of model drugs anchored to the surface of the gold. In this study, gold nanoparticles were not utilized for direct disease treatment but rather as an indirect player in the drug-delivery mechanism. The particular nanoparticles selected were nanorods with a high resonance efficiency and thermal resonance at an 808 nm NIR wavelength. The use of this wavelength avoids tissue and aqueous absorption peaks allowing the use of higher laser powers and good penetration into deeper tissue areas, thereby expanding the selection of target tissues and disease types. One potential problem with magnetically actuated systems may be low magnetic potentials and poor device functioning in deeper tissues using permanent external magnets. 

The polymers PDMS and EVA were selected for use in this bilayer system because the polymers are both biocompatible and have very different thermal expansion coefficients. Initially, bonded films of PDMS and EVA were manufactured and bending could be observed using thermal-conductive heating, ensuring a possible mechanism for diaphragm-bending on a device. Gold nanoparticles were distributed in the PDMS polymer because this polymer cures without solvent evaporation allowing for good nanoparticle distribution in the fluid–solid phase polymerization transition. The solvent evaporation method of EVA film production with the use of DCM solvent would cause great technical problems for producing homogenous gold nanoparticle-EVA films. The effective heating of the PDMS–gold films under NIR laser illumination was observed when a dry membrane was seen to increase over 20 °C in temperature almost instantaneously using an inferred thermal-detection system. When placed under full water immersion, the PDMS film heated quickly by almost 25 °C, reaching an equilibrium with a 30 °C rise within approximately 2 min (Figure 4). Furthermore, when a fully assembled device was placed in an NIR laser beam, the diaphragm heated by over 20 °C almost instantly, with longer exposures resulting in 50 °C increases (Figure 5 and Figure 6). These data confirmed the practicality of the proposed heating mechanism, allowing for rapid heating in an aqueous environment without damage to the polymers or diaphragm. 

When dry assembled devices were illuminated in NIR laser light (rapid heating), the membrane could be visually observed to deflect. This deflection may be seen in Figure 6 but it was not possible to measure this due to the reservoir interfering with optical microscope quantification. Therefore, a device was manufactured with the film oriented in the reverse direction (PDMS uppermost) so that, on heating, the deflection might be visible above the reservoir. A strong convex deflection was observed using this device, as seen in Figure 5, and quantified. These heating and bending observations provided evidence of a functional NIR-actuated device. When a drug-loaded device was illuminated in an aqueous environment and drug release measured, 3 different devices all worked well. They provided 49, 69 and 115 ng/min rates of drug release per heating cycle with highly reproducible doses on sequential actuations (Figure 8 and Figure 9 and Table 1).

The devices provided very similar drug-release profiles when the same device was tested on three subsequent days (e.g., see data for Device A in Figure 8). These μm levels of membrane deflection and nanogram levels of drug release are similar to those observed with magnetically actuated systems [15,16,17,18]. Interestingly, magnetic deflections require very close proximity of the external magnet to the device so that deflection and drug release drop off dramatically at distances greater than 1 cm, which may affect disease treatment in certain locations [15,16,17,18]. It is not known how NIR laser light will be attenuated as a function of tissue depth, but this method of actuation may offer an alternative drug-release mechanism, especially as thin optic fibres may be introduced easily through syringe-needle apertures or catheter lines to overcome tissue-depth problems. 

To treat proliferative diseases, the drug docetaxel works at 10 ng/mL levels, so clearly these levels of drug release into 30 μL of water provide huge excesses of drug for the local treatment of disease, and similar release systems have been shown to provide excess local anti-cancer concentrations [16,17]. Since the device contains almost mg levels of dry drug, the devices have excellent payload capacities for long-term drug release. Obviously, miniaturization may reduce the levels of drug release. 

Overall, this report describes the successful assembly and operation of pilot NIR laser-actuated devices on the bench, and further studies are required with operational devices in vivo.

## Figures and Tables

**Table 1:** Release rate of docetaxel from three different devices.

Sample	Average Release Rate during Actuation (ng/min)	Average Release Rate during Diffusion (ng/min)
Sample A	115.9 ± 23.3	5.65 ± 3.80
Sample B	46.34 ± 26.2	2.93 ± 1.95
Sample C	69.22 ± 22.2	2.20 ± 1.91
